# Gut microbiota analysis in diabetic mice with periodontitis

**DOI:** 10.3389/fmicb.2026.1777180

**Published:** 2026-06-24

**Authors:** Qianjia Pan, Yujie Gu, Minzhe Zhang, Mengfang Jin, Chenyang Shi, Qiuyue Zhang, Bin Wei, Nan Hu, Min Gu

**Affiliations:** 1 Department of Stomatology, The Third Affiliated Hospital of Soochow University, Changzhou, China; 2 School of Medicine, Soochow University, Suzhou, China; 3 College of Pharmaceutical Science & Collaborative Innovation Center of Yangtze River Delta Region Green Pharmaceuticals, Zhejiang University of Technology, Hangzhou, China; 4 Department of Pharmacy, The Third Affiliated Hospital of Soochow University, Suzhou, China; 5 Department of Stomatology, The First People’s Hospital of Changzhou, Changzhou, China

**Keywords:** 16S rDNA sequencing analysis, diabetes mellitus, diabetic periodontitis mice, gut microbiota, periodontitis

## Abstract

**Objectives:**

This study aimed to investigate the composition and alterations of gut microbiota in mice with comorbid diabetes and periodontitis.

**Materials and methods:**

A total of 40 six-week-old male db/db and db/m mice were divided into four groups (*n* = 10 per group): healthy control group, periodontitis group, diabetes group, and diabetes with periodontitis group. Fasting blood glucose (FBG) and oral glucose tolerance test (OGTT) were measured in all four groups to confirm the reliability of the diabetes model. Periodontitis was induced by silk thread ligation, and alveolar bone resorption was assessed using micro-computed tomography (Micro-CT) scanning. Fecal samples were collected from the four groups of mice, and the composition and structure of the gut microbiota were analyzed using 16S ribosomal DNA (16S rDNA) sequencing. Simultaneously, blood samples were collected and centrifuged to obtain serum for the measurement of blood biochemical parameters and inflammatory cytokines.

**Results:**

Compared with the db/m group, the db/m + PD group showed no significant differences in fasting blood glucose and the area under the oral glucose tolerance test curve (AUC). In contrast, compared with the db/db group, the db/db + PD group exhibited significantly elevated fasting blood glucose and the area under the oral glucose tolerance test curve. Relative to the db/m group, the db/m + PD group demonstrated a significant increase in C-reactive protein (CRP). Compared with the db/db group, the db/db + PD group showed high density lipoprotein cholesterol (HDL-C) significantly reduced and Interleukin-10 (IL-10) significantly increased. Pancreatic histology revealed marked islet morphological alterations in both db/db and db/db + PD groups. Significant alveolar bone loss was observed in db/m + PD and db/db + PD groups, with pronounced inflammatory infiltration on periodontal histology, most severe in db/db + PD group. Alpha and beta diversity analyses indicated notable changes in microbial richness and community structure across the four groups. The gut microbiota dysbiosis index was significantly higher in the three experimental groups than in the db/m group. Intergroup comparisons revealed extensive compositional differences at both phylum and genus levels.

**Conclusion:**

Our results showed that the degree of alveolar bone resorption and the microbial dysbiosis index (MDI) in the db/db + PD group were significantly higher than those in the db/m, db/m + PD, and db/db groups. Meanwhile, the destruction of pancreatic *β* cells and periodontal tissues was most severe in the db/db + PD group, and the structure and richness of the gut microbiota in this group were also markedly different from those in the other three groups. Diabetes and periodontitis exerted synergistic effects, inducing and exacerbating gut microbiota dysbiosis in mice, which was closely associated with abnormal blood glucose levels, periodontal inflammation, and alveolar bone resorption. These findings suggest that the gut microbiota may serve as a critical target mediating the bidirectional interaction between diabetes and periodontitis.

## 1 Introduction

Periodontitis is a chronic oral inflammation caused by factors such as microorganisms, leading to the loss of attachment of periodontal supporting tissues, with gingival inflammation and alveolar bone resorption as the main features. In severe cases, teeth may become loose and fall out (Bendek et al., 2021). Diabetes is a chronic disease characterized by high blood sugar due to insufficient insulin secretion or insulin insensitivity (Luong et al., 2021), and it is divided into type 1 diabetes (T1DM) and type 2 diabetes (T2DM). Among them, T2DM accounts for about 90% of all diabetic patients, with the core features being insulin resistance and abnormal *β*-cell function, which seriously affects people’s quality of life (Młynarska et al., 2025). Both periodontitis and type 2 diabetes are chronic inflammatory diseases with high prevalence rates, and there is a clear bidirectional association between them: diabetes aggravates periodontal inflammation and bone resorption, while periodontitis further deteriorates blood sugar control and the overall inflammatory state (Salhi and Reners, 2022; Mata-Monterde et al., 2024; Mirnic et al., 2024).

Previous studies have explained the interaction mechanism between the two from the perspectives of inflammation, immunity, and oxidative stress, but the understanding of the key mediating pathways is still insufficient. The intestine may be an important site for immune regulation in the body and is involved in systemic inflammatory responses (Li et al., 2020). The intestinal microbiota is mainly composed of *Firmicutes*, *Bacteroidetes*, *Proteobacteria* and *Actinobacteria* (Schloissnig et al., 2013). Regarding diabetes, previous studies have pointed out that some intestinal microbiota, such as *Akkermansia muciniphila*, can reduce pro-inflammatory markers and protect the integrity of the intestinal barrier, thereby reducing the risk of diabetes (Iatcu et al., 2021). In addition, short-chain fatty acids (SCFAs) produced by the intestinal microbiota can exert anti-inflammatory effects by inhibiting inflammatory cytokines and thereby alleviating the chronic inflammatory response in diabetes (Baars et al., 2024). A new study used a high-fiber diet specifically designed for the structure of the intestinal microbiota and demonstrated that it could improve glucose homeostasis in T2DM patients, lower pro-inflammatory cytokine levels, and simultaneously change the composition of the intestinal microbiota, increase the diversity of the community, increase the proportion of beneficial bacteria, protect the intestinal barrier function, and provide protection to the host (Chen et al., 2023). Similarly, in periodontitis, Koliarakis et al. (2019) summarized that oral pathogenic bacteria can enter the intestine through continuous swallowing of saliva and other means, and can also spread to the intestine through the bloodstream. These bacteria can change the intestinal microenvironment in the biofilm environment of periodontal disease, which is conducive to the growth of oral pathogenic bacteria, kill beneficial bacteria, damage the intestinal mucosa, induce immune responses, and thereby aggravate the chronic inflammatory response of the host. The latest review also reported a similar conclusion that periodontitis can disrupt the normal composition of oral microbiota, thereby causing intestinal microbiota dysbiosis, and the root cause may be the spread or ectopic colonization of oral microbiota, etc.(Lam et al., 2023). Periodontitis can increase the proportion of *Bacteroidetes* in the intestinal microbiota, reduce the level of *Firmicutes*, cause microbiota dysbiosis, and also reduce the level of intestinal microbiota metabolite linoleic acid (LA), causing an imbalance in Th17/Treg cells in the host and aggravating colonic inflammation (Jia et al., 2024). Studies have found that the intestinal microbiota composition of periodontitis patients after periodontal treatment has undergone significant changes, and the characteristics are close to those of healthy controls (Baima et al., 2024), indicating that periodontitis and its treatment are closely related to the intestinal microbiota. More importantly, the gut microbiota can serve as a link between diabetes and periodontitis. Possible pathways include: Firstly, both diseases can cause intestinal microbiota imbalance, exacerbating the chronic inflammatory state throughout the body; Secondly, the gut microbiota and its metabolites can induce immune responses, leading to blood sugar disorders and periodontal inflammation; finally, the oral-gut axis enables the mutual transportation of microbiota, promoting the mutual influence between periodontitis and diabetes.

Currently, the characteristic changes of the gut microbiota in the state of diabetes mellitus combined with periodontitis, as well as the correlation between gut dysbiosis and disease severity, remain unclear. In this study, db/db mice were employed as diabetic models, with db/m mice serving as normal controls. Experimental periodontitis and diabetic-periodontitis models were established by silk ligation around periodontal tissues. Fecal samples were collected and subjected to 16S ribosomal DNA (16S rDNA) sequencing technology to analyze the gut microbiota composition. Combined with the detection of blood glucose, alveolar bone resorption, inflammatory and biochemical indices, this research aimed to explore the synergistic effects of diabetes and periodontitis on gut microbiota, and reveal the mediating role of gut microbiota in the interaction between the two diseases. This provides experimental evidence for developing preventive and therapeutic strategies targeting gut microbiota.

## 2 Materials and methods

### 2.1 The mice used in the experiment

Twenty specific-pathogen-free (SPF) male db/db mice aged 6 weeks, with the strain background of BKS.Cg-Dock7m+/+Leprdb and genetic background C57BLKS/J, were selected as the spontaneous type 2 diabetes mellitus model. Another 20 specific-pathogen-free (SPF) male db/m mice aged 6 weeks, with the strain background of BKS.Cg -Dock7m+/+ Leprdb and genetic background C57BLKS/J, were included as the normal control group. All of these mice were purchased from Changzhou Carvens Laboratory Animal Co., Ltd. [SCXK (Su) 2021-0013], with the quality certificate number being No. 320730240100378754. The mice were raised in the barrier facility provided by Jiangsu Kebiao Medical Technology Group Co., Ltd. [SYXK (Su) 2021-0013]. The facility environment was automatically controlled by a central air conditioning system, with a temperature range of 20–25 °C, a relative humidity range of 40–70%, a ventilation frequency of 10–15 times/h with fresh air, and a 12-h light/dark cycle. Daily, the animals were provided with sufficient pellet feed and fresh drinking water. The feed was the conventional laboratory SPF-grade mouse food (main components: 10% water, 18% crude protein, 4% crude fat, 5% crude fiber, 8% crude ash, and various amino acids, vitamins and minerals), provided by Jiangsu Kebiao Medical Technology Group Co., Ltd.; both the feed and drinking water were sterilized, and the drinking bottle was sterilized by high-temperature and high-pressure once a week. The animal experiment was carried out after approval by the Ethics Committee of Jiangsu Kebiao Medical Technology Group Co., Ltd., and the ethics approval number was IACUC25-0069.

### 2.2 Experimental grouping

A total of 40 mice were included in this experiment. The normal group consisted of 10 db/m mice (db/m mice), the diabetes model group consisted of 10 db/db mice (db/db mice), the periodontal inflammation group consisted of 10 db/m + PD mice (db/m mice with periodontal ligament ligation), and the diabetes-periodontal inflammation group consisted of 10 db/db + PD mice (db/db mice with periodontal ligament ligation).

### 2.3 Mouse blood sugar test

After obtaining the db/db mice and db/m mice, the mice were quarantined and given adaptive feeding. After the start of the experiment, each mouse was marked using an ear tag. Before and after the period of periodontitis modeling, blood samples were collected from both groups of mice through the tail vein (12 h of fasting before blood collection, no water restriction). The mouse tails were disinfected with alcohol cotton balls and air-dried naturally. Blood samples from the tail tips were collected using a blood collection needle, and fasting blood glucose (FBG) concentrations were measured using the Blood glucose meter and matching blood glucose test strips (ACCU-CHEK Active, Roche Diagnostics GmbH, Switzerland). After all FBG measurements were completed, 50% glucose water solution (prepared by mixing 2 g/kg of anhydrous glucose solid with normal saline) was gavaged to the 4 groups of mice, with a 1-min interval for each mouse, and blood glucose concentrations were measured at 0.5 h, 1 h, 1.5 h, and 2 h after gavage. Oral glucose tolerance (OGTT) analysis was conducted. The changes in blood glucose levels were observed to evaluate the glucose metabolism ability of the mice. If the blood glucose concentration was <7.8 mmol 2 h after OGTT analysis, it indicated that the mice had normal glucose regulation ability, while ≥11.1 mmol indicated that the mice might have diabetes.

### 2.4 Establishment of periodontal disease model

After undergoing quarantine and adaptive feeding, periodontal inflammation modeling was conducted in the second week. Among the established approaches for periodontitis modeling, the ligature-induced periodontitis model presents distinct advantages and is particularly suitable for investigating alveolar bone resorption. One day prior to periodontal ligation, all mice were subjected to a 12 h overnight fasting period with food deprivation, while free access to drinking water was maintained throughout the period (Marchesan et al., 2018). Intraperitoneal injection of 1.5% Pentobarbital sodium (Xi’an Bohua Pharmaceutical Co., Ltd., China) (0.1 mL/20 g) was given for general anesthesia. Using precise surgical instruments, 5-0 silk thread (Nantong Huali Kang Medical Devices Co., Ltd., China) was passed through the space between the first and second molars and the second and third molars of the mice, and the silk thread was firmly tied to the second molar. The ligation was checked every 3 days to see if it had come off. If the ligated thread fell off, it was re-tied. After 4 weeks of modeling, the experimental periodontal inflammation model was successfully induced. The criteria for successful modeling: The mouse gingival tissue was observed to have congestion, bleeding, swelling, pus discharge, ulceration, and erosion, and deep periodontal pockets could be felt upon probing.

### 2.5 Collection and preservation of fecal microbiota

After the periodontitis model was established, the feces of the four groups of mice were collected. The mice were placed alone in sterilized mouse cages for 12 h. The feces of the mice were collected using sterile forceps and placed in sterile test tubes, which were then stored at −80 °C for future examination.

### 2.6 Blood collection

Rat tails were disinfected with alcohol swabs and left to air dry. Blood was collected from the tail tip using a lancet and transferred into 1.5 mL EP tubes. After complete clotting, samples were centrifuged at 3000 rpm and 4 °C for 15 min to harvest serum. The collected serum was aliquoted into new 1.5 mL EP tubes using a pipette, and stored at −80 °C pending further analyses.

### 2.7 Alveolar bone resorption distance

The mice were placed on an experimental table, and the maxilla was removed using an 11th-sized blade and scissors following mouth opening. The mesiodistal boundaries were defined from the mesial aspect of the first molar to the distal aspect of the third molar, with the buccal side limited to the gingival sulcus and the palatal side extending to the palatal midline. The harvested alveolar bone specimens were fixed in 4% paraformaldehyde for 24 h. Micro-computed tomography (Micro-CT) (Model: Quantum GX, United States PerkinElmer Company) scanning was subsequently performed, followed by three-dimensional image reconstruction and analysis. The reconstructed images were processed and analyzed at a resolution of 25 μm using 3D Slicer software (version 5.2). The scanning parameters were set as follows: voltage of 70 kVp, current of 114 μA, pixel size of 15.6 μm, planar pixel resolution of 2048 × 2048, slice spacing of 0.5 mm, and exposure time of 280 ms. Alveolar bone loss was quantified by measuring the distance from the cemento-enamel junction (CEJ) to the alveolar bone crest (ABC) at the mesiodistal center of the first molar on both sides of the maxilla. Each measurement was performed three times, and the average value was recorded as the CEJ-ABC distance for each specimen.

### 2.8 Detection of blood biochemical and inflammatory cytokine markers

Blood biochemical markers including CRP, total cholesterol (TC), triacylglycerol (TG), HDL-C, and low density lipoprotein cholesterol (LDL-C) were measured using a fully automated Biochemical Analyzer (Beckman Coulter AU5800, China), with parameter settings and calibration performed strictly according to the respective kit instructions. CRP was quantified using Immunoturbidimetric Assay Kit (Beijing Leadman Biochemistry Co., Ltd., China), TC was measured via the Cholesterol Oxidase–Peroxidase (CHOD-POD) Assay Kit (Beijing Leadman Biochemistry Co., Ltd., China), TG via the Glycerol-3-phosphate oxidase–phenol + aminophenazone (GPO-PAP) Assay Kit (Beijing Leadman Biochemistry Co., Ltd., China), HDL-C via Direct Method—Selective Inhibition Assay Kit (Beijing Leadman Biochemistry Co., Ltd., China), and LDL-C via the Direct Method—Surfactant Clearance Assay Kit (Beijing Leadman Biochemistry Co., Ltd., China). After centrifugation, the supernatant serum was determined for eight cytokines including TNF-α, IL-1β, IL-2, IL-4, IL-6, IL-8, IL-10 and IFN-γ by Flow Cytometric Luminometer iMatrix 100 (JOINSTAR, China) using an 8-Plex Cytokine Detection Kit (Chongqing Biocentury Biotech Co., Ltd., China).

### 2.9 Hematoxylin–eosin (HE) staining

After fixing the collected left maxillary bone and pancreatic samples in 4% neutral paraformaldehyde for 24 h (the jawbone tissue needs to be decalcified with 10% EDTA for 3-4 weeks, with the decalcification solution changed every 3 days), the tissue is dehydrated using a tissue processing instrument (Thermo Fisher Company, United States) in a gradient, embedded in paraffin via embedding machine (Leica Company, Germany), and then sectioned at a thickness of 4 μm. Appropriate sections are selected, deparaffinized and hydrated with xylene and gradient ethanol, stained with HE, sealed with neutral resin, and observed and photographed under a electron microscope (Nikon Company, Japan).

### 2.10 Bacterial genomic DNA extraction and 16S rDNA Illumina sequencing

According to the manufacturer’s protocol, the total DNA of fecal microorganisms was extracted using DNA extraction kit (Shanghai Meiji Biomedical Technology Co., Ltd., China). The extracted genomic DNA was detected by JY600C electrophoresis instrument (Beijing Junyi Dongfang Electrophoresis Equipment Co., Ltd., China). The V3-V4 region of the bacterial 16S ribosomal DNA (16S rDNA) gene was amplified using the GeneAmp 9,700 thermal cycler (Applied Biosystems, United States), with primers 338F (5′-ACTCCTACGGGAGGCAGCAG-3′) and 806R (5′-GGACTACHVGGGTWTCTAAT-3′). The PCR products were purified and recovered using the AxyPrep DNA gel recovery kit (AP-MN-P-500, Axygen, United States). The fecal microbiota structures were evaluated on the Genetic sequencing analyzer (Illumina Miseq, United States) through dual-index amplification and sequencing, and then QIIME (version 1.6.0) bioinformatics analysis was performed.

### 2.11 Calculation of the microbial dysbiosis index (MDI)

The Microbial Dysbiosis Index (MDI) indicates the extent of gut microbial dysbiosis. Higher MDI scores reflect more severe disruption to the intestinal microbiota. The MDI is calculated as follows: MDI = log (sum of the abundances of gut microbiota increased in the disease group/sum of the abundances of gut microbiota decreased in the disease group).

### 2.12 Data processing

The data were statistically analyzed and visualized using SPSS software (Version 26.0, Chicago, United States), GraphPad Prism software (Version 9.2.0, California, United States), and R-studio software (version 3.3.1, United States). Bioinformatics analysis was conducted using QIIME (version 1.6.0, United States), and 3D Slicer (Version 5.2, United States) was used for 3D image reconstruction and analysis. The results were expressed as mean ± standard deviation for normally distributed variables, or median (first quartile to third quartile) for non-normally distributed variables. For normally distributed data, one-way analysis of variance (ANOVA) was used to compare differences among the four groups. For non-normally distributed data, the Kruskal–Wallis *H* test was applied. The Bray-curtis distance algorithm was employed in the Principal Coordinates Analysis (PCoA). The difference test was performed using a two-tailed test and False Discovery Rate (FDR) for multiple test correction. *p* < 0.05 indicated statistically significant differences.

### 2.13 Methods for correlation analysis and functional prediction

We used Spearman correlation analysis, which assessed correlations based on the ranked values of two variable sets. It can clarify the association between independent and dependent variables, as well as the degree of influence of the former on the latter. Briefly, raw data of both groups are sorted in ascending or descending order and converted into corresponding ranks. The correlation is then calculated using these ranked data. Microbial functional profiles were predicted using the Phylogenetic Investigation of Communities by Reconstruction of Unobserved States 2 (PICRUSt2) pipeline based on 16S rRNA gene sequencing data. Briefly, amplicon sequence variant representative sequences were aligned to a reference phylogenetic tree to infer unobserved gene content and gene family copy numbers. Predicted functional profiles were annotated against the Kyoto Encyclopedia of Genes and Genomes (KEGG) database to obtain KEGG orthology terms and metabolic pathway information. Functional prediction reliability was evaluated using the nearest sequenced taxon index, and only high-confidence functional results were retained for subsequent pathway analysis. Then we applied Principal component analysis (PCA) to explore the clustering pattern across all samples and visualize intergroup differences.

## 3 Results

### 3.1 Changes in blood glucose-related indicators before and after periodontitis modeling

**Figure 1:**
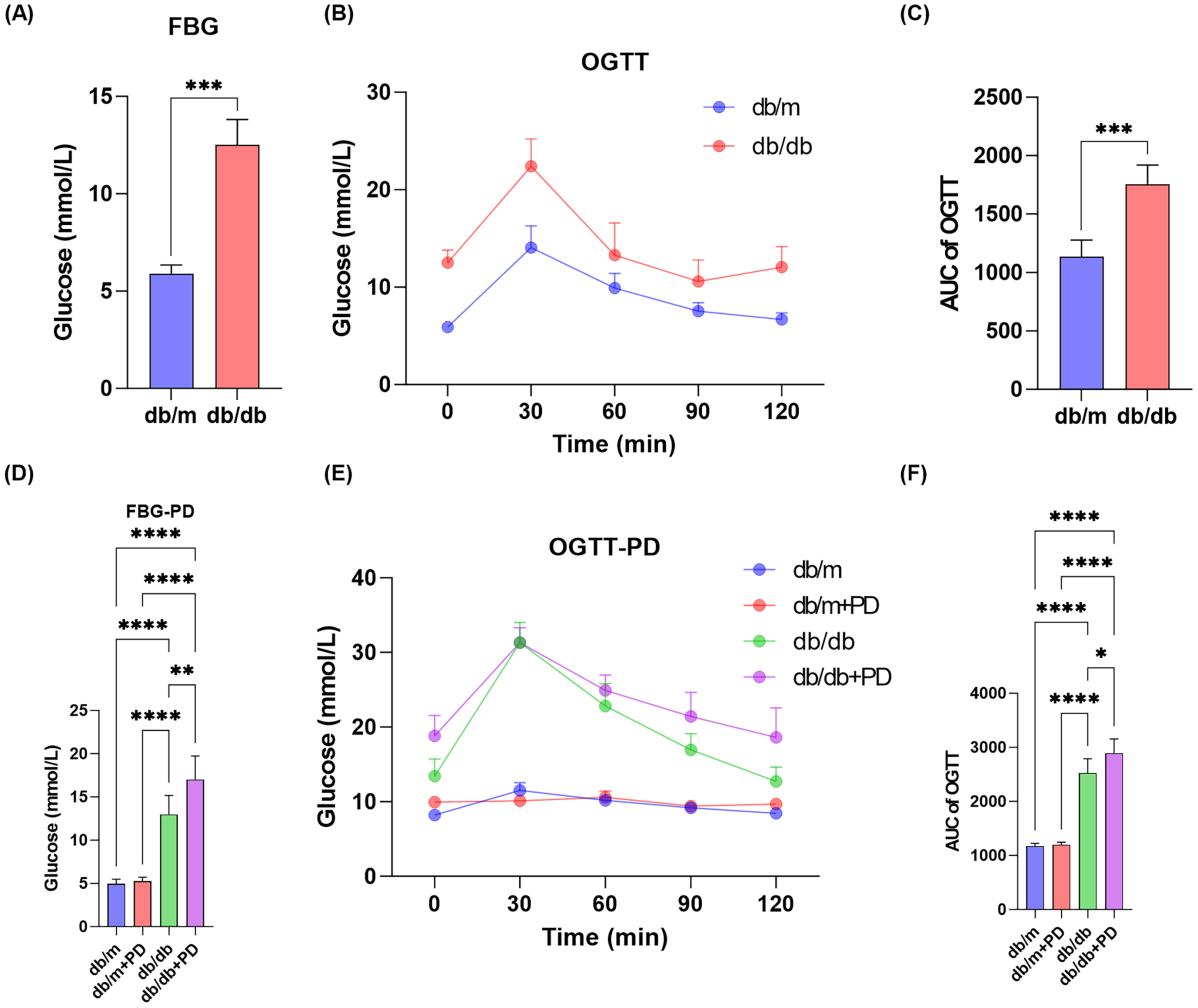
Measurement of fundamental blood glucose parameters in the four groups of mice. Fasting blood glucose and oral glucose tolerance test were measured in mice of all four groups before and after periodontitis modeling. These measurements were used to validate the establishment of the type 2 diabetic mouse model and the status of control groups, followed by intergroup statistical comparisons. **(A)** FBG after a 12-hour fast in diabetic and non-diabetic mice before periodontitis modeling. **(B)** OGTT after a 12-hour fast in diabetic and control mice before periodontitis modeling. **(C)** Differences in the area under the OGTT curve after a 12-hour fast among groups before periodontitis modeling. **(D)** FBG after a 12-hour fast in the four groups of mice 4 weeks after periodontitis modeling; **(E)** OGTT after a 12-hour fast in the four groups of mice 4 weeks after periodontitis modeling. **(F)** Differences in the area under the OGTT curve after a 12-hour fast among the four groups 4 weeks after periodontitis modeling. Statistical analysis was performed based on the area under the curve in panel **B**, and the results are presented in panel **C**. *p< 0.05, **p < 0.01, ***p < 0.001, ****p < 0.0001 by two-tailed one-way ANOVA FBG, fasting blood glucose; OGTT, oral glucose tolerance test; ANOVA, analysis of variance.

Fasting blood glucose (FBG) and oral glucose tolerance test (OGTT) were measured in all four groups of mice before and after periodontitis modeling to verify the establishment of the type 2 diabetes mouse model and the reliability of the control groups. It was found that before the experiment, db/db mice exhibited FBG and 2-h postprandial OGTT results ≥11.1 mmol/L, whereas db/m mice showed FBG and 2-h postprandial OGTT results <11.1 mmol/L (Figures 1A,B). Differences in the area under the OGTT curve after a 12-hour fast among groups before periodontitis modeling (Figures 1C). After the completion of periodontitis modeling, both the db/db and db/db + PD groups displayed FBG and 2-h postprandial OGTT results ≥11.1 mmol/L, while the db/m and db/m + PD groups maintained FBG and 2-h postprandial OGTT results <11.1 mmol/L (Figures 1D,E). Compared with the db/m group, the db/m + PD group showed no significant differences in FBG or the area under the OGTT curve (AUC). In contrast, compared with the db/db group, the db/db + PD group exhibited significantly elevated FBG and OGTT AUC (Figures 1D,F). These results indicate that the presence of periodontitis does not significantly affect FBG or OGTT in healthy mice. However, in diabetic mice, periodontitis markedly increases FBG and OGTT, suggesting that periodontitis alone does not impact blood glucose or glucose tolerance levels in mice, but under diabetic conditions, it further elevates blood glucose levels, exacerbates glucose intolerance, and exhibits a synergistic effect. Additionally, the results demonstrate that db/db gene-induced mice serve as a reliable and stable model for type 2 diabetes, while mice in the db/m group can be considered a dependable control.

### 3.2 Results of blood biochemistry and inflammatory factor indices in the four groups of mice

Table 1 details the blood biochemistry and inflammatory factor indices in the four groups of mice. Compared with the db/m group, the db/m + PD group showed a significant increase in CRP. Compared with the db/db group, the db/db + PD group exhibited a significant decrease in HDL-C and a significant increase in IL-10. Although CRP showed a decreasing trend, the difference was not statistically significant (*p* > 0.05). Additionally, levels of IL-6, IL-8, and IL-1β were elevated but did not reach statistical significance. These results indicate that periodontitis exerts different effects on blood biochemical indices in healthy mice versus diabetic mice. This suggests that the systemic inflammatory state induced by periodontitis is altered under diabetic conditions, implying that both diseases may exert their pathogenic effects by influencing the concentrations of CRP and HDL-C.

**Table 1:** Inflammatory factors and blood biochemical indicators in the four groups of mice.

Parameters	db/m (*n* = 10)	db/m + PD (*n* = 10)	db/db (*n* = 10)	db/db + PD (*n* = 10)	*p*-values
CRP (mg/L)	3.94 ± 3.036^a^	9.927 ± 4.657^c^	6.9 ± 6.113	3.5 ± 2.337	0.004**
TC (mmol/L)	3.069 ± 0.317^bc^	3.008 ± 0.200^bc^	3.888 ± 0.301	4 ± 0.417	<0.001***
TG (mmol/L)	0.791 ± 0.185	0.65 (0.53~0.73)^b^	0.956 ± 0.263	0.671 ± 0.172	0.024*
HDL-C (mmol/L)	2.495 ± 0.191^bc^	2.597 ± 0.272^bc^	3.56 ± 0.345^c^	3.082 ± 0.385	<0.001***
LDL-C (mmol/L)	0.477 ± 0.063^c^	0.454 ± 0.047^c^	0.545 ± 0.089	0.84 (0.80~0.89)	<0.001***
IL-2 (pg/mL)	1.694 ± 0.202	1.453 ± 0.087^b^	1.62 (1.52~2.82)	1.623 ± 0.199	0.024*
IL-4 (pg/mL)	1.506 ± 0.403	1.51 (1.33~1.68)	1.380 ± 0.172	1.379 ± 0.157	0.505
IL-6 (pg/mL)	2.016 ± 0.177	1.950 ± 0.149	2.170 ± 0.151	2.190 ± 0.321	0.072
IL-10 (pg/mL)	2.8 ± 5.949^c^	0.61 (0.56~0.65)	0.567 ± 0.078^c^	0.72 (0.66~3.09)	0.016*
TNF-α (pg/mL)	0.45 ± 0.028	0.45 (0.32~0.46)	0.444 ± 0.059	0.491 ± 0.046	0.121
IFN-γ (pg/mL)	1.619 ± 0.520	1.169 ± 0.424	2.27 ± 1.294	2.135 ± 1.219	0.115
IL-1β (pg/mL)	0.429 ± 0.085	0.459 ± 0.078	0.437 ± 0.059	0.495 ± 0.079	0.346
IL-8 (pg/mL)	12.16 (10.83~13.90)	9.845 ± 1.517	9.69 (9.11~12.89)	11.32 ± 3.418	0.139

Non-normal distribution data are expressed by the median (quartile) method. Db/db mice were diabetes group; Db/db + PD was diabetes periodontitis group; Db/m mice were used as the normal control group; Db/m + PD was periodontitis group. *p*-values calculated using one-way analysis of variance or rank sum test, or Chi-squared test. *p* < 0.05 was statistically significant, *p < 0.05, **p < 0.01, ***p < 0.001. ^a^*p* < 0.05 vs. db/m + PD group; ^b^*p* < 0.05 vs. db/db group; ^c^*p* < 0.05 vs db/db + PD group. CRP, C-reactive protein; TC, total cholesterol; TG, triacylglycerol; HDL-C, high density lipoprotein cholesterol; LDL-C, low density lipoprotein cholesterol; IL-2, Interleukin-2; IL-4, Interleukin- 4; IL-6, Interleukin-6; IL-10, Interleukin-10; TNF-α, Tumor necrosis factor-alpha; IFN-γ, Interferon- gamma; IL-1β, Interleukin-1β; IL-8, interleukin-8.

### 3.3 Results of HE staining of pancreatic tissue in the four groups of mice

**Figure 2:**
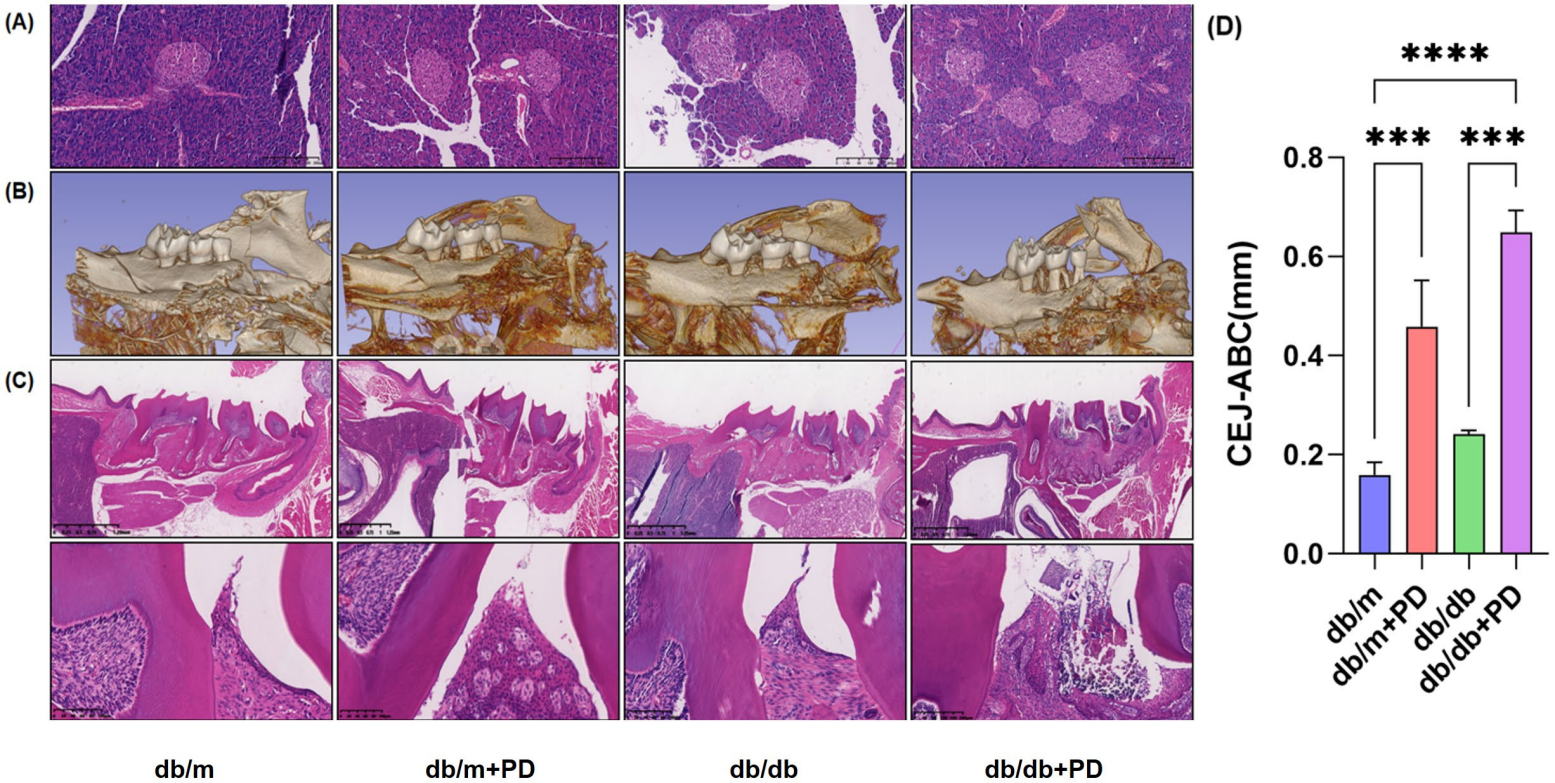
HE staining of pancreatic and periodontal tissues, micro-CT images of alveolar bone and the distance from the cemento-enamel junction (CEJ) to the alveolar bone crest (ABC) in the four groups of mice. HE staining was performed on pancreatic tissues and left maxillary alveolar bone samples collected from all four groups of mice. Micro‑CT scanning was applied to quantify the extent of alveolar bone resorption in each experimental group. **(A)** Results of HE staining of pancreatic tissue in the four groups of mice. **(B)** Three-dimensional reconstructed images of the left maxillary alveolar bone in the four groups of mice obtained by Micro-CT. **(C)** HE staining of the left maxillary alveolar bone in the four groups of mice. In the three sets of images **(A–C)**, from left to right, they correspond sequentially to db/m, db/m + PD, db/db and db/db + PD. **(D)** Comparison of alveolar bone resorption distance in four groups of mice. *p < 0.05, **p < 0.01, ***p < 0.001, ****p < 0.0001 by two-tailed one-way ANOVA. Micro-CT, Micro-computed tomography.

The pancreatic HE staining (Figure 2A) showed that in the db/m group, the islet cells were arranged densely and regularly, with a clear islet boundary. The cell morphology was normal, the nuclei were uniform in size with evenly distributed chromatin, and the cytoplasm was lightly stained pale pink. In contrast, in the db/m + PD group, the overall pancreatic islet structure was largely preserved; however, the cell arrangement appeared slightly looser, with a modest increase in intercellular spacing. Localized mild cellular edema was observed, and the nuclear chromatin appeared somewhat loosely distributed. These results indicate that periodontitis may induce a mild systemic inflammatory response, exerting a certain degree of impact on the pancreatic islets, though the damage was relatively slight. In the db/db group of mice, the pancreatic islets were markedly atrophic, with disordered cell arrangement. Some islet cells exhibited degeneration and necrosis, manifested as nuclear pyknosis, fragmentation, and vacuolar degeneration. The number of cells within the islets was reduced, indicating that under diabetic conditions, the structure and function of pancreatic *β*-cells were significantly impaired, leading to insulin secretion dysfunction and consequently an imbalance in blood glucose regulation. In the db/db + PD group, the islet architecture was severely disrupted, with exacerbated atrophy and highly irregular morphology. Cell arrangement was extremely disordered, accompanied by extensive cell degeneration and necrosis, along with prominent inflammatory cell infiltration. The function of the islets was severely compromised. These findings suggest that when periodontitis and diabetes coexist, the destruction of pancreatic β-cells is more severe. This indicates that periodontitis and diabetes act synergistically, further exacerbating β-cell damage, intensifying the inflammatory state, and severely impairing islet function, thereby causing serious disturbances in the body’s blood glucose regulation and metabolic functions.

### 3.4 Micro-CT assessment of alveolar bone resorption in the four groups of mice

Four weeks after ligation with silk sutures, macroscopic observation of the left maxillary second molar in the mice revealed that, compared with the db/m group, the db/m + PD and db/db + PD groups exhibited obvious gingival margin edema, partial erosion, bleeding, and poor adaptation to the tooth surface. To verify the reliability of the experimental periodontitis model in mice and to investigate the effects of diabetes and periodontitis on alveolar bone resorption, Micro-CT scanning and three-dimensional alveolar bone reconstruction were performed for each group (Figure 2B). The results showed that, compared with the db/m group, the db/m + PD group displayed significant alveolar bone resorption with mild furcation exposure. Compared with the db/db group, the db/db + PD group exhibited more severe alveolar bone resorption, reaching approximately half of the root length, accompanied by aggravated bone loss adjacent to the tooth and increased furcation exposure. Moreover, the distance from the cementoenamel junction (CEJ) to the apical bone crest (ABC) of the second molar in the maxilla of the db/m + pd. group and the db/db + pd. group of mice was significantly greater than that of the db/m group and the db/db group (*p* < 0.001). Meanwhile, the CEJ-ABC distance of the db/db + pd. group was higher than that of the db/m + pd. group, but this difference did not reach statistical significance (Figure 2D). Therefore, we conclude that diabetes exacerbates alveolar bone resorption induced by periodontitis, predisposes to furcation exposure, and intensifies the destructive effects of periodontitis on bone tissue.

### 3.5 Morphology of periodontal tissues in the four groups of mice

In the db/m group of mice, the periodontal tissue exhibited a clear structure, a uniformly thick epithelial layer, evenly distributed cells within the connective tissue, and regularly arranged fibers, with no obvious inflammatory cell infiltration observed. The alveolar bone morphology was normal. Compared to the db/m group, the periodontal tissue in the db/m + PD group showed disruption of gingival epithelial continuity, extensive inflammatory cell infiltration, disordered arrangement of periodontal ligament fibers, and visible alveolar bone resorption. In the db/db group, the epithelial layer of the periodontal tissue was slightly thinner, with somewhat disordered cell arrangement and relatively sparse fibrous tissue. A small number of inflammatory cells were scattered throughout, and the alveolar bone density was reduced, although the degree of resorption was relatively mild. In the db/db + PD group, the periodontal tissue displayed alternating hyperplasia and atrophy of the epithelial layer, along with massive and extensively diffuse infiltration of inflammatory cells. The inflammatory area was more widespread compared to the other three groups. Severe alveolar bone resorption and a significant reduction in bone height were observed. Therefore, periodontitis alone increases the number of inflammatory cells, induces an inflammatory state in the periodontal tissues, leads to alveolar bone resorption, and damages the periodontal structures. When periodontitis coexists with diabetes, the hyperglycemic environment and chronic inflammatory state of diabetes further exacerbate the susceptibility to and severity of periodontitis, thereby aggravating alveolar bone loss and causing severe damage to the periodontal tissues (Figure 2C). This finding is consistent with the observations from Micro-CT.

### 3.6 Results of gut microbiota sequencing analysis

#### 3.6.1 Microbial dysbiosis index (MDI) and alpha diversity analysis

A total of 40 fecal samples were sequenced. After optimization, the gut microbiota yielded 2,510,104 high-quality sequences with a total base count of 1,060,137,387. The average sequence length was 422 bases. Sequences were rarefied based on the smallest sample sequence count, and only operational taxonomic units (OTUs) with at least 5 sequences present in a minimum of 3 samples were retained. This resulted in the identification of 11 phyla, 14 classes, 33 orders, 52 families, 117 genera, 206 species, and a total of 518 OTUs.

**Figure 3:**
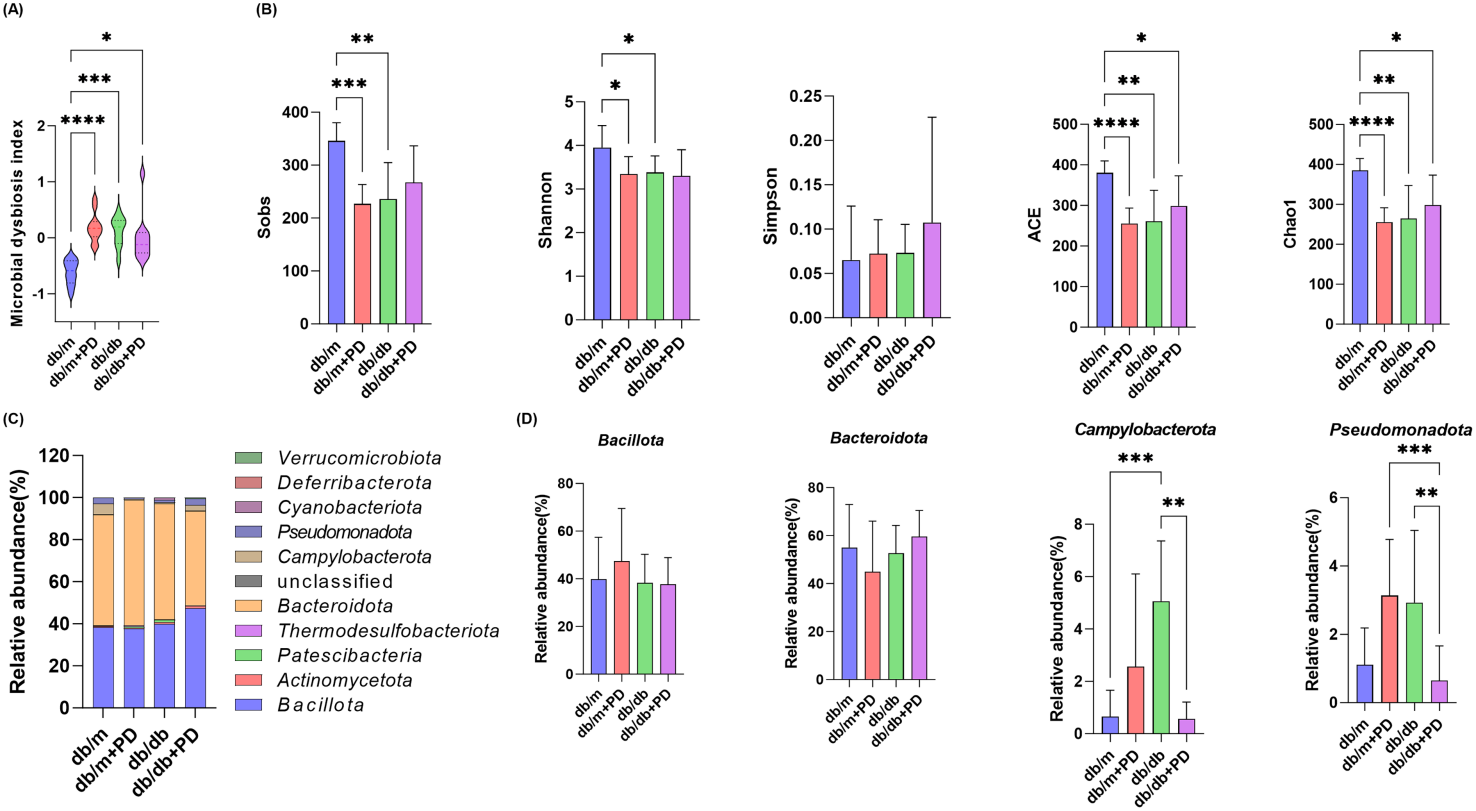
Analysis of microbial dysbiosis index (MDI), alpha diversity and phylum-level species composition of the gut microbiota across the four groups. The gut microbiota dysbiosis index was analyzed in four groups to evaluate the community structure and dysbiosis status of intestinal microbiota. Alpha diversity analysis was adopted to assess the richness and diversity of gut microbial communities. Cluster analysis was further performed to identify the predominant bacterial phyla at the phylum level among the four groups. **(A)** Microbial dysbiosis index of gut microbiota in four groups of mice. **(B)** Alpha diversity analysis. **(C)** Bar plot analysis of gut microbiota composition at the phylum level. **(D)** Dominant bacterial phyla in the gut microbiota at the phylum level.*p < 0.05, **p < 0.01, ***p < 0.001, ****p < 0.0001 by two-tailed one-way ANOVA.

The microbial dysbiosis index (MDI) quantifies the degree of microbial ecological imbalance, with a higher value indicating greater disruption of the microbiota (Figure 3A). The MDI in the db/m + PD group was significantly higher than that in the db/m group (*p* < 0.001), indicating that periodontitis induces structural dysbiosis in the intestinal microbiota of mice. Compared with the db/db group, the MDI in the db/db + PD group showed an increasing trend, although the difference did not reach statistical significance (*p* > 0.05). The MDI in the db/db group was significantly higher than that in the db/m group (*p* < 0.001), demonstrating that diabetes also leads to intestinal microbiota dysbiosis in mice. Notably, the db/db + PD group exhibited the highest MDI among all four groups, suggesting that the coexistence of diabetes and periodontitis exacerbates the impact on the intestinal microbiota, resulting in severe disruption of the gut microbiota structure.

Alpha diversity analysis was employed to evaluate the richness and diversity of the intestinal microbiota in mice. Significant variations in alpha diversity indices indicated marked alterations in the richness and evenness of the gut microbial community among the four groups. The Sobs, ACE, and Chao1 indices were primarily used to assess species richness. Among these indices, Sobs refers to the number of species actually observed and directly reflects species richness. ACE is an abundance-based coverage estimator of species richness. Chao1 is an estimator of the total number of species in a sample based on an extrapolation method. While the Shannon and Simpson indices were applied to measure species diversity. Among these indices, Shannon is a diversity index that comprehensively considers both species richness and evenness, while Simpson is used to measure the dominance of predominant species within a community, representing the probability that two sequences drawn randomly from a sample belong to the same species. No significant differences were observed in the Simpson index among the four groups (*p* > 0.05), indicating comparable community diversity across groups. Compared with the db/m group, the db/m + PD group showed significant decreases in the Sobs, Shannon, ACE, and Chao1 indices. This suggests that the intestinal microbiota of mice with periodontitis had significantly lower richness and diversity than that of healthy mice. Periodontitis reduced the richness and diversity of the normal intestinal microbiota, indicating a decrease in beneficial bacteria and an increase in pathogenic bacteria, leading to an abnormal gut microbiota structure. Compared with the db/db group, the db/db + PD group exhibited increased values in the Sobs, Simpson, ACE, and Chao1 indices; however, these differences were not statistically significant (*p* > 0.05) (Figure 3B). This indicates that the diabetic state influences the effect of periodontitis on the richness and diversity of the intestinal microbiota in mice, with both diseases jointly affecting the gut microbiota.

#### 3.6.2 Phylum-level composition analysis of the gut microbiota

Through clustering, the relative abundance of microbial communities in the samples was obtained at different taxonomic levels. At the phylum level of the gut microbiota, the main dominant phyla across the four groups of mice were *Bacillota*, *Bacteroidota*, C*ampylobacterota*, and *Pseudomonadota* (Figures 3C,D). Compared with the db/m group, the db/m + PD group showed an increase in *Bacillota*, *Campylobacterota* and *Pseudomonadota*, while *Bacteroidota* decreased, however, none of these differences reached statistical significance. Compared with the db/db group, the db/db + PD group exhibited a significant reduction in *Campylobacterota* and *Pseudomonadota*. Additionally, the db/db group showed a notably higher abundance of *Campylobacterota* than the db/m group. These results indicate that the diabetic state can alter the changes in gut microbiota composition induced by periodontitis, and that both diseases influence the normal composition of the intestinal microbiota.

#### 3.6.3 Beta diversity analysis and genus-level composition analysis of the gut microbiota

**Figure 4:**
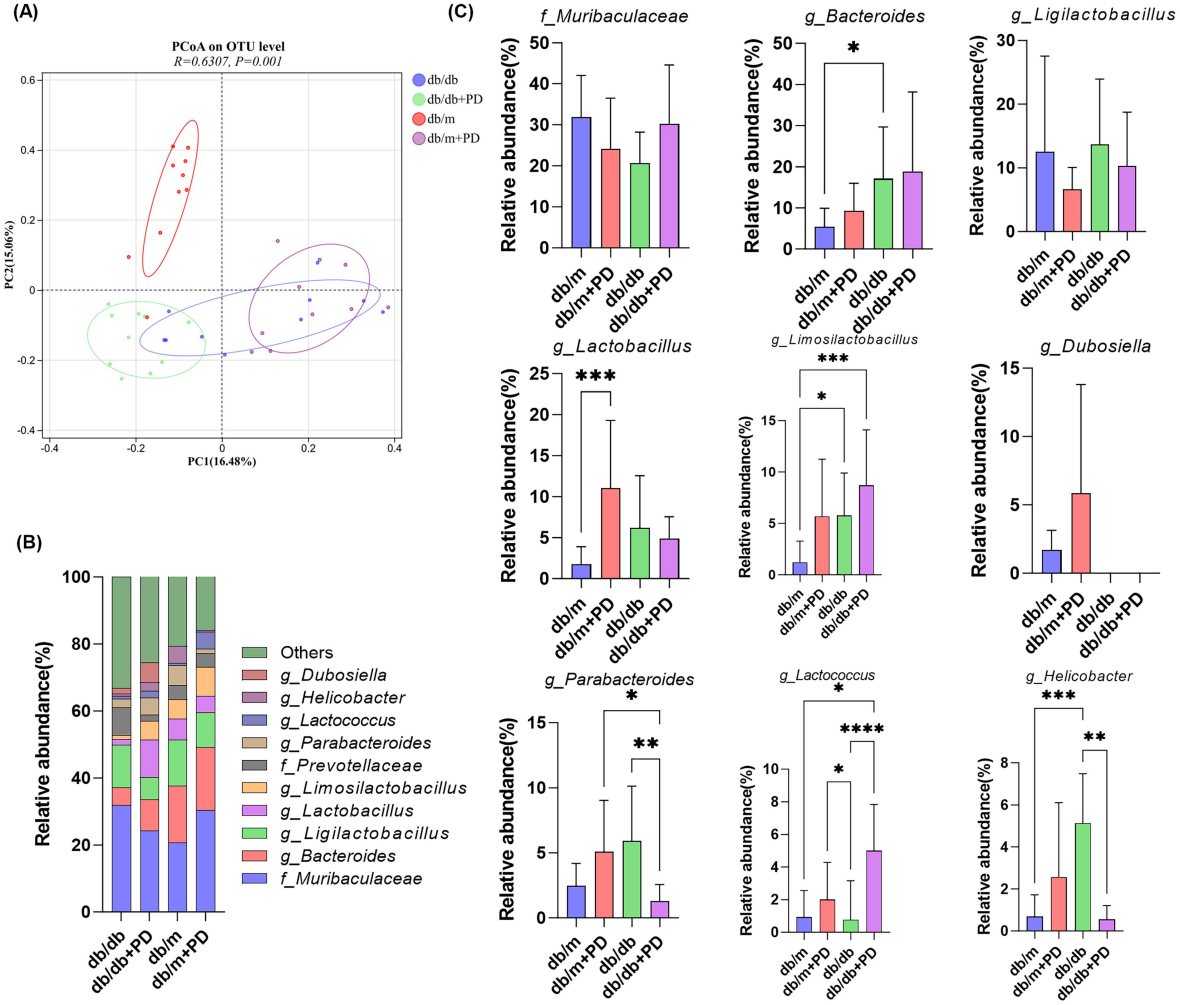
Beta diversity analysis and genus-level compositional analysis of the gut microbiota. Beta diversity analysis was performed to evaluate the differences in gut microbial community composition among the four groups. Cluster analysis was subsequently applied to identify the dominant bacterial genera at the genus level across all groups. **(A)** Principal Coordinates Analysis based on Bray-Curtis distance at the operational taxonomic unit (OTU) level, statistical analysis and visualization were conducted using R language (Version 1). *R* = 0.6307, *p* = 0.001. Each group (db/m, db/m + PD, db/db, db/db + PD) included 10 samples. **(B)** Bar plot analysis of gut microbiota composition at the genus level. **(C)** Dominant bacterial genera in the gut microbiota at the genus level. *p < 0.05, **p < 0.01, ***p < 0.001, ****p < 0.0001 by two-tailed one-way ANOVA.

Beta diversity reflects overall differences in community composition and structure between samples. It is used to evaluate whether experimental groups exhibit distinct gut microbiota profiles, with greater separation indicating more significant divergence in microbial community structure. In this study, principal coordinate analysis (PCoA) was performed on the gut microbiota of four groups of mice at the OTU level based on Bray-Curtis distance. Statistical differences were observed in the gut microbiota composition among the four groups (*R* = 0.6307, *p* < 0.001), indicating that the between-group differences were greater than the within-group differences, and these differences were significant (*p* < 0.05). The first principal component (PC1) and the second principal component (PC2) explained 16.48 and 15.06% of the total variation, respectively (Figure 4A). Pairwise Analysis of Similarities (ANOSIM) was performed to determine significant differences in microbiota structure between paired experimental groups. Pairwise ANOSIM further verified that all pairs of groups exhibited significant separation (*p* < 0.001). Notably, the db/m group displayed the strongest separation from the db/db group (*R* = 0.722) and the db/m + PD group (*R* = 0.714). Table 2 details *p*-values from pairwise comparisons of *β*-diversity of gut microbiota among the four groups. These results demonstrate that both diabetes and periodontitis significantly affect the structure of the gut microbiota. Diabetes induces notable changes in the gut microbiota structure of mice with periodontitis, while periodontitis also alters the gut microbiota of diabetic mice.

**Table 2:** Pairwise ANOSIM analysis of gut microbial β-diversity among the four groups.

Comparison	*R* value	*p*-value
db/db vs. db/db + PD	0.473	0.00033 < 0.001
db/db vs. db/m	0.722	0.00016 < 0.001
db/db vs. db/m + PD	0.530	0.00019 < 0.001
db/db + PD vs. db/m	0.662	0.00011 < 0.001
db/db + PD vs. db/m + PD	0.688	0.00007 < 0.001
db/m vs. db/m + PD	0.714	0.00014 < 0.001

β-diversity was determined based on Bray-Curtis distance. Pairwise comparisons were performed using ANOSIM, and *p* < 0.05 was considered statistically significant.

At the genus level of the intestinal microbiota, the main dominant genera in the four groups of mice were *f_Muribaculaceae*, *g_Bacteroides*, *g_Ligilactobacillus*, *g_Lactobacillus*, *g_Limosilactobacillus*, *g_Dubosiella*, *g_Parabacteroides*, *g_Lactococcus*, and *g_Helicobacter* (Figure 4B). Compared with the db/m group, the db/m + PD group showed a significant increase in *g_Lactobacillus* (*p* < 0.001), while *g_Limosilactobacillus*, *g_Dubosiella*, *g_Parabacteroides*, *g_Lactococcus* and *g_Helicobacter* also exhibited an increasing trend, though these changes were not statistically significant (*p* > 0.05). Compared with the db/db group, the db/db + PD group demonstrated a significant decrease in *g_Parabacteroides* and *g_Helicobacter*, along with a significant increase in *g_Lactococcus* (Figure 4C). These results indicate that under the combined condition of periodontitis and diabetes, the intestinal microbiota shows an increase in *g_Lactococcus* and a decrease in *g_Parabacteroides* and *g_Helicobacter*.

#### 3.6.4 Differential abundance analysis of species at the phylum and genus levels in the gut microbiota

**Figure 5:**
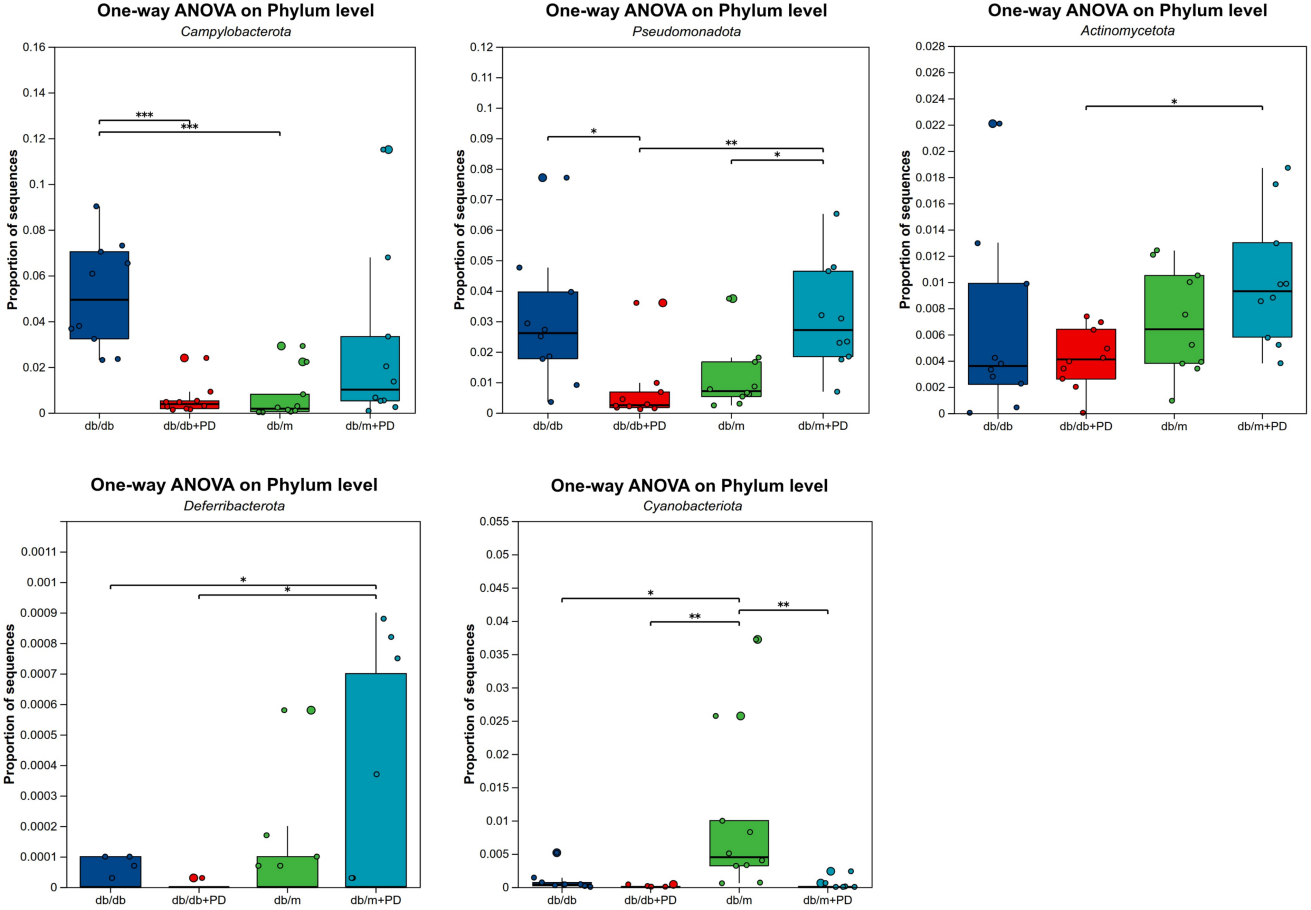
Differential abundance analysis of gut microbiota at the phylum level. One-way analysis of variance (ANOVA) was performed to compare the relative abundance of gut microbiota among the four groups at the phylum level. *p < 0.05, **p < 0.01, ***p < 0.001 by two-tailed one-way ANOVA.

**Figure 6:**
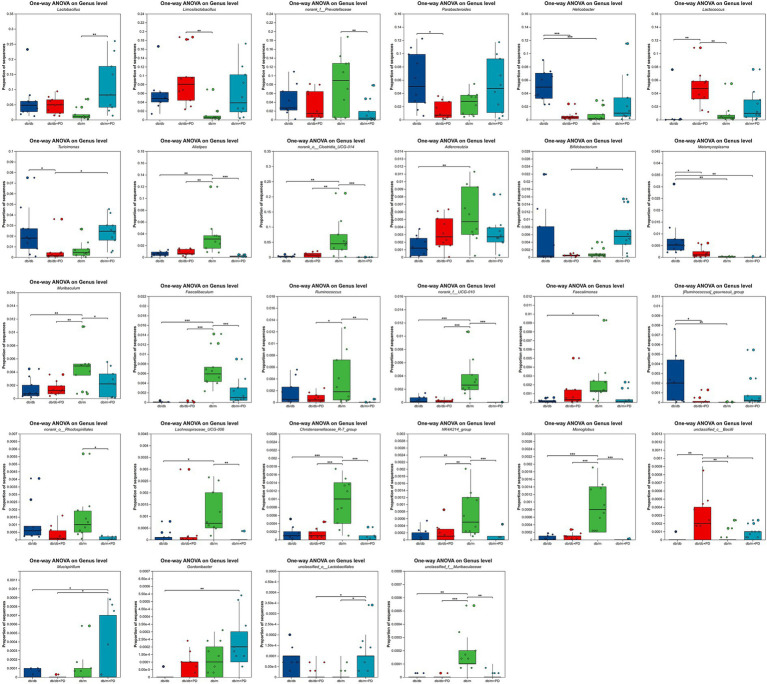
Differential abundance analysis of gut microbiota at the genus level. One-way analysis of variance (ANOVA) was performed to compare the relative abundance of gut microbiota among the four groups at the genus level. *p < 0.05, **p < 0.01, ***p < 0.001 by two-tailed one-way ANOVA.

One-way analysis of variance (ANOVA) confirmed statistically significant intergroup differences in microbial abundance at multiple taxonomic levels. ANOVA was performed on the abundance of 11 bacterial phyla and 117 bacterial genera in the gut microbiota of the four mouse groups to compare their abundance across the groups. The results revealed significant between-group differences in 5 phyla (Figure 5) and 28 genera (Figure 6), with statistical significance.

To investigate the impact of diabetes on the gut microbiota, we first compared db/db mice with db/m mice. Significant differences were observed at the phylum level for *Campylobacterota* and *Cyanobacteriota* (Figure 5), while at the genus level, significant differences were found in the abundance of 15 genera: *Helicobacter*, *Alistipes*, *norank_o__Clostridia_UCG-014*, *Faecalibaculum*, *Adlercreutzia*, *Metamycoplasma*, *Muribaculum*, *norank_f__UCG-010*, *Faecalimonas*, *[Ruminococcus]_gauvreauii_group*, *Lachnospiraceae_UCG-006*, *Christensenellaceae_R-7_group*, *NK4A214_group*, *unclassified_f__Muribaculaceae*, and *Monoglobus* (Figure 6). Subsequently, we compared db/db + pd. mice with db/m + pd. mice and identified significant differences at the phylum level for *Pseudomonadota*, *Actinomycetota*, and *Deferribacterota* (Figure 5). At the genus level, significant differences were observed in nine genera: *unclassified_c__Bacilli*, *Mucispirillum*, *Gordonibacter*, *unclassified_o__Lactobacillales*, *Parabacteroides*, *Lactococcus*, *Turicimonas*, and *Bifidobacterium*. No common differentially abundant microbial taxa were found between the two comparison groups (Figure 6).

Similarly, to investigate the impact of periodontitis on the gut microbiota, we compared db/m + pd. mice with db/m mice. Significant differences in relative abundance were observed at the phylum level for *Pseudomonadota* and *Cyanobacteriota* (Figure 5), while at the genus level, significant differences were identified for 16 genera: *Ruminococcus*, *norank_f__UCG-010*, *norank_o__Rhodospirillales*, *Lachnospiraceae_UCG-006*, *Christensenellaceae_R-7_group*, *NK4A214_group*, *Monoglobus*, *unclassified_o__Lactobacillales*, *unclassified_f__Muribaculaceae*, *Lactobacillus*, *norank_f__Prevotellaceae*, *Turicimonas*, *Alistipes*, *norank_o__Clostridia_UCG-014*, *Bifidobacterium* and *Faecalibaculum* (Figure 6). Next, a comparison between db/db + pd. mice and db/db mice revealed significant differences at the phylum level for *Campylobacterota* and *Pseudomonadota* (Figure 5), and at the genus level for 7 genera: *Parabacteroides*, *Helicobacter*, *Lactococcus*, *Turicimonas*, *Metamycoplasma*, *[Ruminococcus]_gauvreauii_group*, and *unclassified_c__Bacilli* (Figure 6). Through these comparisons, we found that *Pseudomonadota* was the common differentially abundant phylum (Figure 5), and *Turicimonas* was the common differentially abundant genus across both comparison groups (Figure 6). Interestingly, in the comparison between db/m + pd. and db/m mice, both *Pseudomonadota* and *Turicimonas* were significantly increased in db/m + pd. mice. However, in the comparison between db/db + pd. and db/db mice, both taxa were significantly decreased in db/db + pd. mice. This suggests that diabetes may play a modifying role in mice with periodontitis, influencing the effect of periodontitis on the gut microbiota.

Finally, we compared db/db + pd. mice with db/m mice and found that the phylum *Cyanobacteriota* showed a significant difference at the phylum level (Figure 5). At the genus level, significant differences were observed in 13 genera: *Ruminococcus*, *norank_f__UCG-010*, *Christensenellaceae_R-7_*group, *NK4A214_*group, *Monoglobus*, *unclassified_c__Bacilli*, *unclassified_f__Muribaculaceae*, *Limosilactobacillus*, *Lactococcus*, *Alistipes*, *norank_o__Clostridia_UCG-014*, *Muribaculum*, and *Faecalibaculum* (Figure 6). Integrating results from all comparison groups, we found that at the phylum level, the four phyla *Pseudomonadota*, *Cyanobacteriota*, *Campylobacterota*, and *Pseudomonadota* were significantly altered in both periodontitis and diabetes (Figure 5). At the genus level, the following 18 genera were identified as differentially abundant in the context of both periodontitis and diabetes: *norank_f__UCG-010*, *[Ruminococcus]_gauvreauii_group*, *Lachnospiraceae_UCG-006*, *Christensenellaceae_R-7_group*, *NK4A214_group*, *Monoglobus*, *unclassified_c__Bacilli*, *unclassified_o__Lactobacillales*, *unclassified_f__Muribaculaceae*, *Parabacteroides*, *Helicobacter*, *Lactococcus*, *Turicimonas*, *Alistipes*, *norank_o__Clostridia_UCG-014*, *Bifidobacterium*, *Metamycoplasma*, and *Faecalibaculum* (Figure 6).

#### 3.6.5 Correlation analysis of gut microbiota with periodontal indicators, diabetes indicators, inflammatory factors and blood biochemical indicators

**Figure 7:**
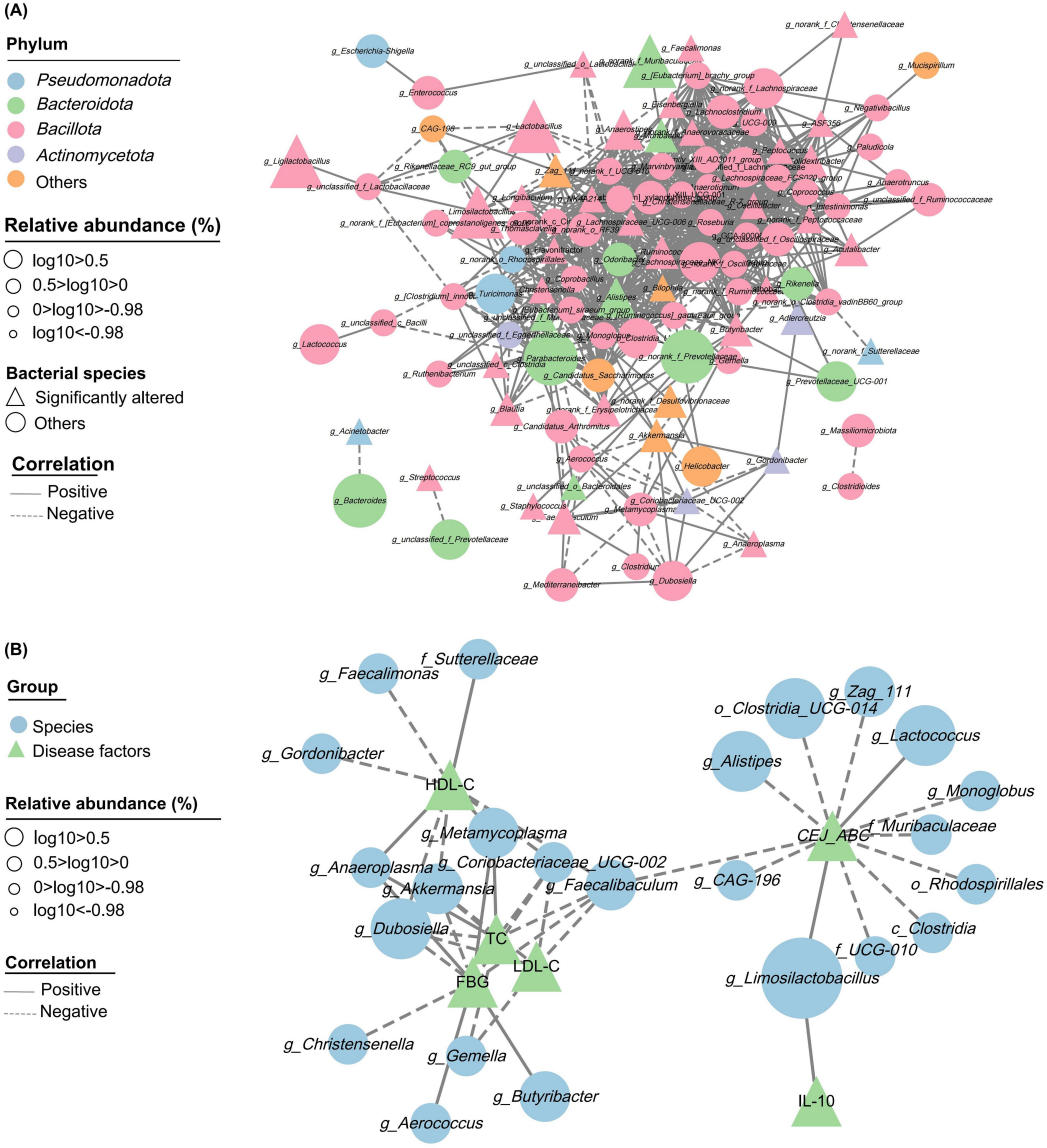
Correlation analysis between gut microbiota and periodontal parameters, diabetic indicators, inflammatory factors, and blood biochemical indices in the four groups of mice. Spearman correlation analysis was performed to explore the correlations of periodontal parameters, diabetic indicators, inflammatory factors and blood biochemical indices with the top 50 abundant bacterial genera in the gut microbiota at the genus level. **(A)** Species abundance of gut microbiota at the genus level. **(B)** Correlation analysis between gut microbiota and relevant indicators.

Spearman correlation analysis was applied to assess directional associations between key gut bacteria and relevant important parameters. We explored the correlations between periodontal indicators, diabetes indicators, inflammatory factors, blood biochemical indicators and the top 50 ranked genera-level gut microbiota. After excluding genera that showed no correlation with these indicators, a total of 37 genera were found to be associated with at least one of the measured parameters (Figure 7). Among them, *Lactococcus* and *Limosilactobacillus* showed a positive correlation with alveolar bone resorption distance, while eight genera, including *Faecalibaculum*, *Zag_111* and *Ruminococcus*, showed a negative correlation with alveolar bone resorption distance. Four genera, including *Butyribacter*, *Metamycoplasma*, and *Clostridium*, were positively correlated with fasting blood glucose levels, whereas five genera, such as *Faecalibaculum*, *Dubosiella*, and *Akkermansia*, were negatively correlated with fasting blood glucose levels. Four genera, including *Dubosiella* and *Akkermansia*, were positively correlated with CRP levels, while five genera, such as *Butyribacter* and *Metamycoplasma*, were negatively correlated with CRP. *Candidatus_Saccharimonas*, *norank_f__UCG-010*, and *norank_o__Clostridia_UCG-014* were positively correlated with TG levels, whereas *Thomasclavelia* was negatively correlated with TG. L*imosilactobacillus*, *Lactobacillus*, *Lactococcus*, and *norank_f__Erysipelotrichaceae* were positively correlated with IL-10 levels, while five genera, including *Akkermansia* and *Zag_111*, were negatively correlated with IL-10. Seven genera, including *Metamycoplasma* and *Clostridium*, were positively correlated with TC levels, whereas 10 genera, such as *Faecalibaculum*, *Dubosiella*, and *Akkermansia*, were negatively correlated with TC. Six genera, including *Metamycoplasma* and *Clostridium*, were positively correlated with HDL-C levels, while nine genera, such as *Faecalibaculum* and *Dubosiella*, were negatively correlated with HDL-C. B*utyribacter*, *Metamycoplasma*, and *Clostridium* were positively correlated with LDL-C levels, whereas six genera, including *Dubosiella*, were negatively correlated with LDL-C.

#### 3.6.6 KEGG analysis in the four groups of mice

**Figure 8:**
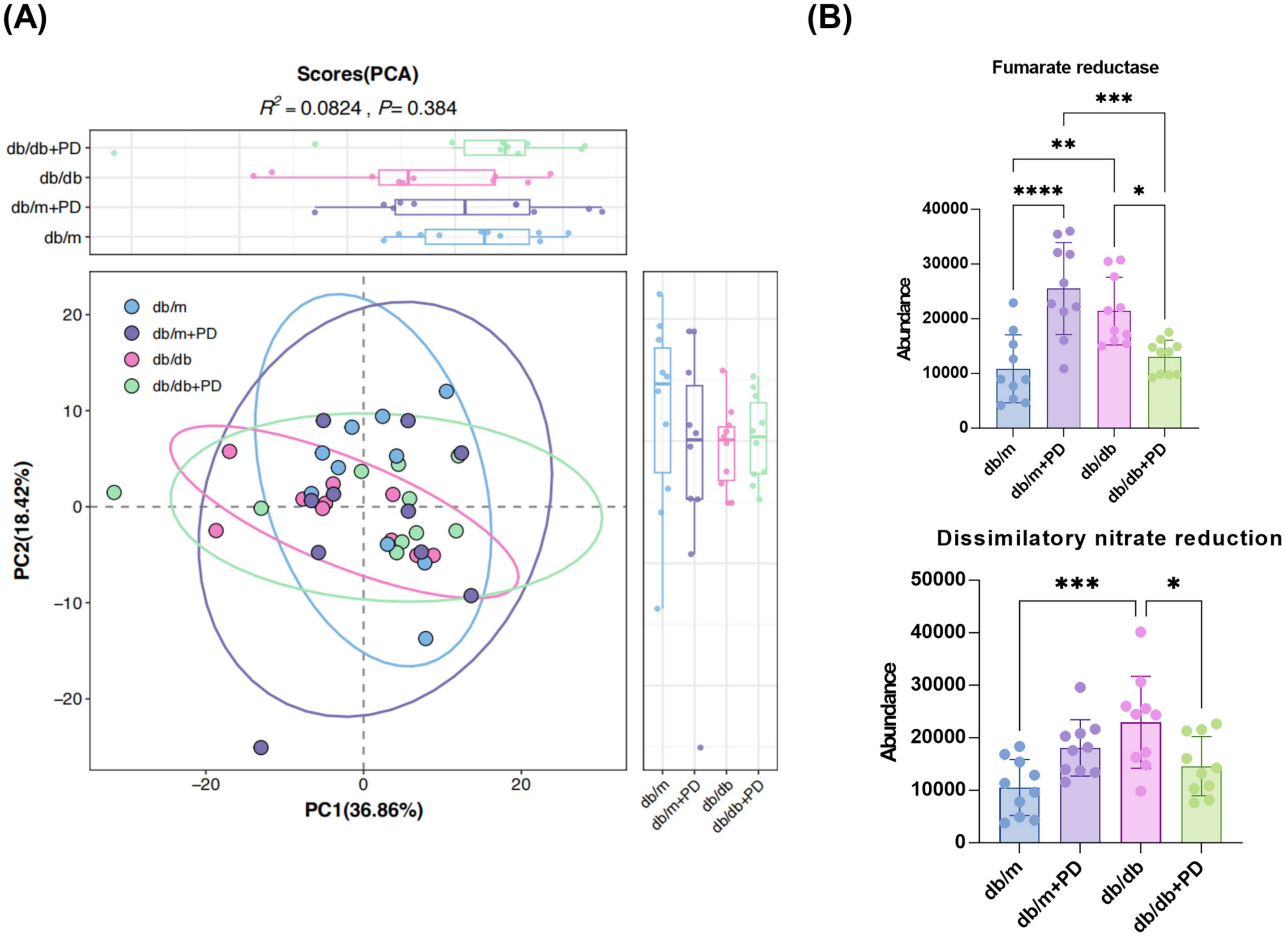
Functional analysis of intestinal microbiota in four groups of mice. Based on the 16S rDNA sequencing data obtained from the four mouse groups, functional prediction of the gut microbiota was conducted using PICRUSt2, further assigned against the KEGG database.Principal component analysis (PCA) was then applied for data screening and result visualization. **(A)** PCA scores of four groups of mice. **(B)** Comparison of fumarate reductase and dissimilatory nitrate reduction across four mouse groups. PICRUSt2,Phylogenetic Investigation of Communities by Reconstruction of Unobserved States 2; KEGG,Kyoto Encyclopedia of Genes and Genomes.

PICRUSt2 was used to predict the functional potential of gut microbiota, and KEGG was applied to annotate metabolic and signaling pathways linked to microbial functions. Based on 16S rDNA sequencing data from four groups of mice, the functional profiles of gut microbiota were predicted using PICRUSt2 and annotated against KEGG database. Subsequently, we performed PCA for data screening and visualized the analytical results. No significant differences were observed in the overall sample distribution. Nevertheless, alterations were detected in several metabolic pathways closely associated with periodontitis, including fumarate reductase and dissimilatory nitrate reduction (Figure 8).

## 4 Discussion

This study found that the IL-10 level in the db/db + PD group was significantly higher than that in the db/db group. Neurath and Kesting (2024) also reported elevated serum IL-10 levels in patients with chronic periodontitis. Additionally, Sumbayak et al. (2023) observed a tendency for higher IL-10 levels in elderly periodontitis patients compared to adult patients, which may be related to periodontal conditions and the severity of inflammation. However, Acharya et al. (2015) reported significantly lower IL-10 levels in patients with periodontitis compared to healthy controls. Therefore, this difference may be related to sample size and the severity of diabetes. Previous studies have indicated elevated CRP levels in patients with chronic periodontitis (Valiyaveetil Karattil et al., 2022). In our study, CRP was significantly higher in the db/m + pd. group compared to the db/m group. Teles et al. (2024) reported similar findings, showing significantly higher CRP levels in periodontitis patients compared to periodontally healthy controls (*p* < 0.05), with a significant decrease in serum CRP 6 months after non-surgical periodontal therapy (NSPT). Furthermore, this study revealed that compared to the db/db group, the db/db + PD group exhibited a significant reduction in HDL-C. Lee et al. (2018) found that women with lower HDL-C levels had a significantly higher risk of developing periodontitis than those with higher HDL-C levels. Similarly, Zhao et al. (2025) demonstrated that periodontal inflammation and its severity were associated with decreased HDL-C.

We found significant differences in gut microbial composition between diabetic and non-diabetic mice at both the phylum and genus levels, consistent with previous research (Ahmad et al., 2019; Sedighi et al., 2017; Han et al., 2019). At the genus level, the proportion of *Helicobacter* in the gut microbiota of db/db mice was significantly higher than in db/m mice, aligning with the findings of Yang et al. (2021), who reported an increased proportion of *Helicobacter* in diabetic groups compared to controls, along with reduced gut microbiota diversity in T2DM groups. Among *Helicobacter* species, *Helicobacter pylori* (*H. pylori*) is considered closely associated with T2DM. A review summarizing previous literature indicated that the prevalence of *H. pylori* is higher in diabetic patients than in non-diabetic individuals, which may be related to elevated glucose levels and alterations in the gastric mucosa (Mansori et al., 2020). Previous studies have suggested that impaired immune function, reduced gastric acid secretion, inflammatory states, and insulin resistance in diabetic patients may be linked to *H. pylori* (Polyzos et al., 2011; He et al., 2014). However, variations in *H. pylori* detection methods and quality can affect measurement accuracy, leading to inconsistent results. Therefore, further high-quality, large-sample studies are needed to clarify the relationship between diabetes and *H. pylori*. Additionally, this study found that the proportions of *Faecalibacterium* and *Bifidobacterium* were significantly lower in diabetic mice compared to non-diabetic mice, supporting earlier findings. James et al. (2022) noted a significant reduction in certain butyrate-producing *Firmicutes* in T2DM patients, with *Faecalibacterium prausnitzii* being a key component. *Firmicutes* are major producers of butyrate, which reduces hepatic glucose production (Tanase et al., 2020), alleviates inflammation, enhances insulin sensitivity, and protects the gut. This highlights the bidirectional relationship between T2DM and gut microbiota dysbiosis. Similarly, Ghaemi et al. (2020) reported comparable findings, showing significantly lower counts of *Faecalibacterium prausnitzii* and *Bifidobacterium* in T2DM patients compared to healthy individuals. This suggests that T2DM is associated with changes in gut microbial composition, though the exact mechanisms and causal relationships remain unclear. In summary, the hyperglycemic environment caused by diabetes significantly affects the composition and relative abundance of the gut microbiota, while gut microbiota dysbiosis is also closely linked to the development of T2DM.

By comparing the gut microbiota composition between mice with periodontitis and those without, combined with analyses of alpha diversity and PCoA, we found significant differences in both gut microbial diversity and compositional structure between the two groups. This is consistent with the findings of Wu et al. (2022), who used 16S rDNA and LC–MS techniques to explore the impact of periodontitis on the gut microbiota in rats and observed significant differences in microbial composition and dominant genera between periodontitis and periodontally healthy groups. In the present study, we found that the proportion of *Ruminococcus* in db/m + pd. mice was significantly lower than in db/m mice. This result is not entirely consistent with previous conclusions. Kato et al. (2018) reported a significant increase in the proportion of *Ruminococcus* in the gut microbiota of mice treated with *Porphyromonas gingivalis* (Pg) compared to sham-treated mice. Meanwhile, Kamer et al. (2024) found through LEfSe analysis that the abundance of gut microbiota such as *Ruminococcus* and *Lactobacillus* was significantly higher in patients with mild periodontitis compared to those with severe periodontitis. Thus, such discrepancies may be related to variations in the severity of periodontal inflammation, experimental methods, and individual differences. *Ruminococcus* can produce short-chain fatty acids (SCFAs), such as acetate via the Wood–Ljungdahl pathway. SCFAs play important roles in maintaining intestinal metabolic homeostasis, modulating intestinal inflammatory states, and regulating immune responses. Their metabolites, primarily butyrate, acetate, and propionate, contribute to maintaining gut homeostasis and alleviating intestinal inflammation (Akhtar et al., 2022). Furthermore, a clinical study found that the abundance of *Lactobacillus* in the gut microbiota of periodontitis patients was reduced compared to the control group (Baima et al., 2024), which differs from our findings and may be attributed to differences in sample size or individual variability. On one hand, *Lactobacillus* can produce lactic acid to inhibit the growth of oral pathogenic bacteria and regulate the oral microecology. On the other hand, it promotes the increase of beneficial gut bacteria, thereby enhancing SCFA production, which helps reduce inflammation and maintain intestinal homeostasis (Chen and Zhang, 2023). Topical application of *Lactobacillus* has been shown to reduce periodontal pocket depth, inhibit alveolar bone resorption, and effectively improve periodontal inflammation (Nguyen et al., 2021).

Current research suggests that periodontitis primarily affects the gut microbiota through two pathways: the oral-gut axis and the hematogenous route. Bao et al. (2022) used 16S rDNA sequencing to analyze fecal and salivary samples from patients with healthy periodontium and those with severe periodontitis. They found significant differences in gut microbiota composition between the two groups, with more saliva-derived microbial structures detected in the severe periodontitis group. This suggests that periodontitis may influence normal gut microbiota structure through salivary microbiota, thereby contributing to gut microbiota dysbiosis. This aligns with the perspective of Sulaiman et al. (2024), who proposed that the oral cavity of periodontitis patients harbors a large number of periodontal pathogens, such as *Porphyromonas gingivalis* (Pg). These oral bacteria can translocate to the gut via the digestive tract, though under normal conditions, most bacteria cannot survive the gastric acid barrier or the effects of bile acids. However, certain oral pathogens, such as Pg, can tolerate the acidic gastric environment, reach the intestines, and colonize, leading to an exaggerated gut response, intestinal microbiota disruption, and systemic inflammatory reactions (Atarashi et al., 2017). This further supports the idea that oral pathogens from periodontitis can enter the gut via the oral-gut axis and induce abnormal changes in gut microbiota. Additionally, periodontitis may also affect the gut microbiota through hematogenous spread. Bacteria and their byproducts can enter the bloodstream via ulcerated periodontal pocket linings. Upon reaching the intestines, they may disrupt the gut microbiota and trigger systemic inflammation (Hajishengallis and Chavakis, 2021).

Diabetes and periodontitis share similarities in their impact on gut microbiota changes. For instance, a reduction in butyrate-producing bacteria has been observed in both conditions. Decreased butyrate synthesis can trigger systemic inflammation, impair glucose metabolism, and elevate blood sugar levels, while an inflammatory state may also induce periodontitis (Chang et al., 2022; James et al., 2022). This suggests that gut microbiota may play a role in the bidirectional relationship between diabetes and periodontitis through this mechanism. Our study found that when periodontitis coexists with diabetes, the severity of periodontal inflammation increases, alveolar bone resorption becomes more pronounced, blood glucose levels further rise, and the inflammatory state is exacerbated, demonstrating a synergistic effect between the two conditions. Previous studies have indicated that oral pathogens and inflammatory factors from periodontitis can enter the gut via hematogenous pathways. One study indicated that excessive IL-17 in periodontal pockets may enter the bloodstream through ulcerated surfaces, activating the nuclear factor-κB (NF-κB) pathway, upregulating inflammatory factors (IL-1β, IL-6, TNF-*α*), inducing insulin resistance, promoting the development of diabetes and further exacerbating the inflammatory state of periodontitis (Abdel-Moneim et al., 2018). Additionally, Pg in periodontitis can increase intestinal permeability, leading to endotoxemia and systemic inflammation, thereby increasing susceptibility to diabetes (Arimatsu et al., 2014). Similarly, diabetes can also increase intestinal permeability, resulting in a low-grade systemic inflammatory state that exacerbates diabetes and promotes the progression of periodontitis (Iatcu et al., 2021). Moreover, studies have found that both conditions reduce gut microbiota diversity, indicating an imbalance in gut microbiota characterized by an increase in pathogenic bacteria and a decrease in beneficial bacteria, which further aggravates gut dysbiosis and promotes the progression of both diseases (Li et al., 2021). Therefore, periodontitis and diabetes can easily induce each other and exert a synergistic effect, exacerbating the severity of both diseases and amplifying the systemic inflammatory state. However, this study has certain limitations. First, the reliance of 16S gene sequencing on specific gene regions limits the identification of species at the genus level, while metagenomic data are crucial for validating results. Additionally, differences in measurement methods for certain microbial groups can lead to varying outcomes, potentially affecting the interpretation of findings. In summary, diabetes and periodontitis can influence each other through the gut microbiota, but the specific mechanisms remain unclear. Future high-quality studies are needed to further elucidate their bidirectional relationship.

## 5 Conclusion

In summary, this study demonstrates that both diabetes and periodontitis disrupt the composition, diversity, and community structure of the gut microbiota in mice. Compared with normal mice, the other three groups exhibited significant gut microbial dysbiosis. Several intestinal microbes, including *Campylobacterota*, *Pseudomonadota* and *Helicobacter*, were associated with both diabetes and periodontitis. Meanwhile, correlation analysis revealed that gut microbiota was closely linked to fasting blood glucose levels, alveolar bone resorption, and inflammatory blood biochemical parameters. These findings suggest that gut microbiota-targeted microbiota intervention, such as the rational application of beneficial bacteria, represents a novel strategy for the treatment of chronic inflammatory diseases including diabetes and periodontitis. Such approaches are expected to become effective therapies and exert positive effects on the prevention and management of these diseases.

## Data Availability

The datasets generated and/or analyzed during the current study are available in the NCBI repository, accession number: PRJNA1337453-https://www.ncbi.nlm.nih.gov/bioproject/PRJNA1337453.
